# Idiopathic thoracic spinal cord herniation into the vertebra progressing for 3 years

**DOI:** 10.1016/j.radcr.2024.02.091

**Published:** 2024-03-19

**Authors:** Motoki Yamataka, Satoshi Tsutsumi, Kasumi Inami, Natsuki Sugiyama, Hideaki Ueno, Masanori Ito, Hisato Ishii

**Affiliations:** Department of Neurological Surgery, Juntendo University Urayasu Hospital, Urayasu, Chiba, Japan

**Keywords:** Spinal cord herniation, Intravertebral herniation, Progressive pathology, Etiology

## Abstract

A 43-year-old, previously healthy man experienced a decreased sensation in the left lower extremity without preceding spinal trauma. At presentation, the patient exhibited slight motor weakness in the left lower extremity, in addition to decreased pain sensation below the ipsilateral T7. Spinal magnetic resonance imaging (MRI) revealed abnormal findings consistent with idiopathic thoracic spinal cord herniation (ITSCH) at the T5/6 level. Computed tomography (CT) revealed a small vertebral erosion at the lower T5. The patient's symptoms gradually progressed over the next 3 years. MRI revealed marked lateral elongation of the cord at the T5/6 and apparent intravertebral cord herniation. The patient underwent ITSCH reduction through T5-6 laminectomies. The herniated cord was vertically long with a bulbous rostral part. Successful ITSCH reduction was achieved and the patient's postoperative course was uneventful. ITSCH is a progressive pathology that requires prompt surgical reduction. Certain ITSCHs may be complicated by intravertebral cord herniation.

## Introduction

Idiopathic thoracic spinal cord herniation (ITSCH) is a rare defect of the ventromedial or mediolateral dura mater associated with herniation of the spinal cord. To date, approximately 350 cases have been reported [1]. Although the etiology of ITSCH remains unknown, congenital causes involving ventral dura mater duplication, pre-existing pseudomeningocele, and transdural appendix of the spinal cord, in addition to inflammation, degeneration, spinal trauma, bone spur, and thoracic disc herniation, have been postulated to be associated factors [2], [3], [4], [5], [6], [7]. Also, transdural intravertebral spinal cord herniation is known to be a rare form of ITSCH [8]. Patients with ITSCH require reduction of the herniation and subsequent widening of the dura defect as early as possible for common progressive myelopathy [1,[9], [10], [11]]. Typically, patients with ITSCHs are managed by posterior approach through laminectomy, while an anterior thoracotomy approach has been adopted for selected cases [12].

Here, we present a unique case of progressive ITSCH presenting with progressive intravertebral herniation for 3 years.

## Case report

**Fig. 1:**
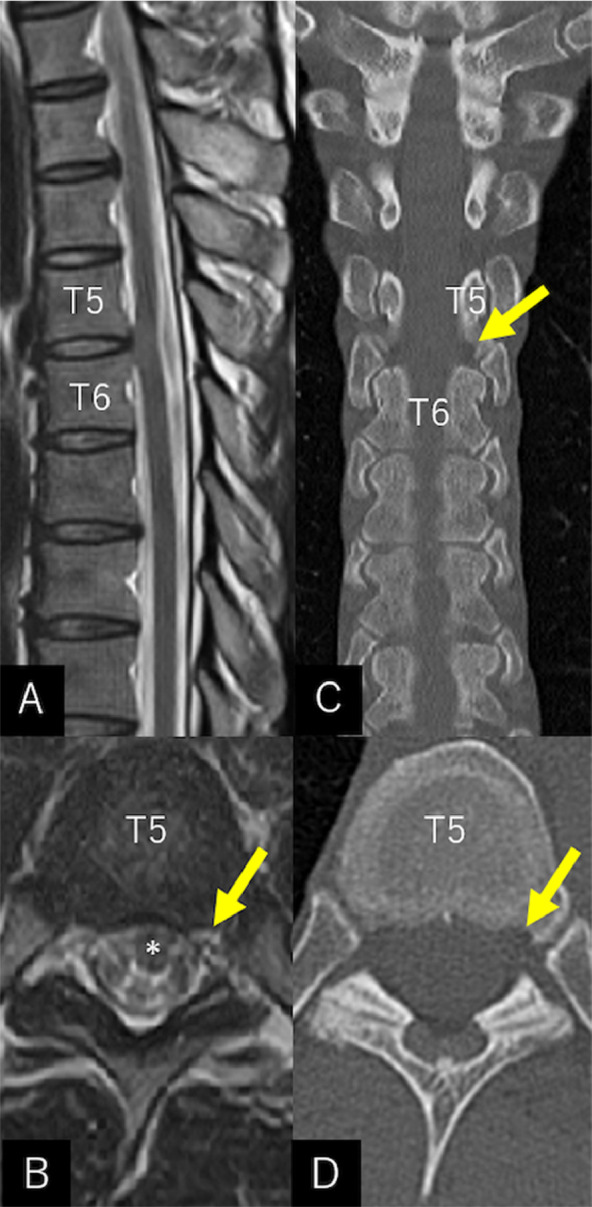
Sagittal (A) and axial (B) T2-weighted magnetic resonance imaging performed at the initial presentation showing a localized, ventral displacement of the spinal cord at the T5/6. On coronal (C) and axial (D) computed tomography scans, a well-circumscribed, small vertebral erosion is identified at the lower T5, just above the left facet joint (*arrow*). The vertebral erosion appears hyperintense on T2-weighted sequence (B***,*** arrow). *Asterisk:* spinal cord.

**Fig. 2:**
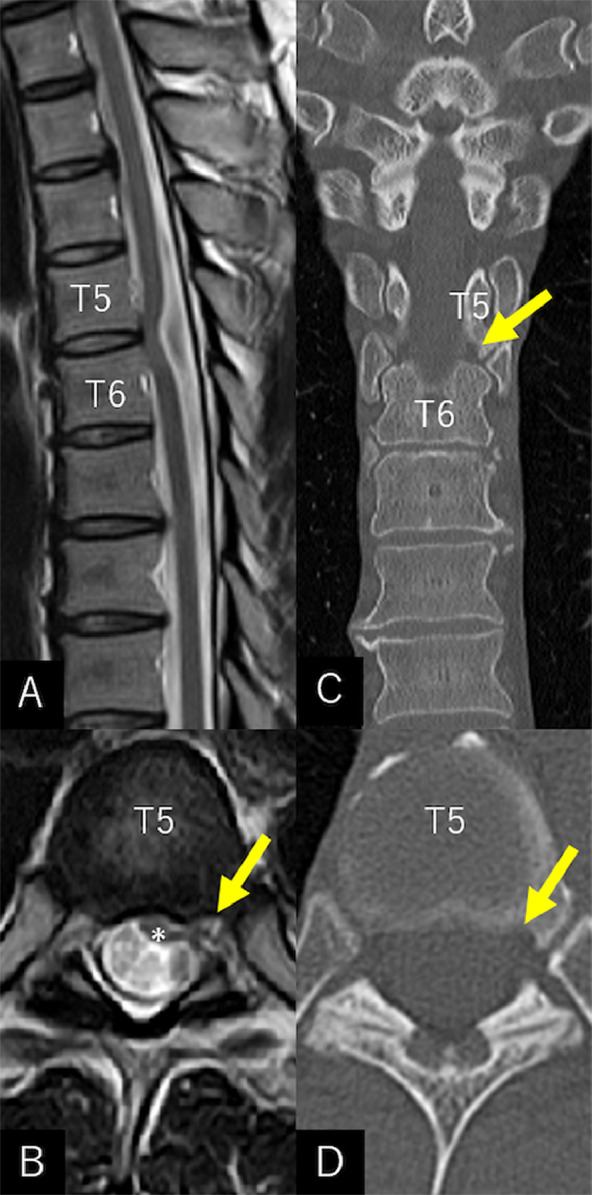
Sagittal (A) and axial (B) T2-weighted magnetic resonance imaging performed 39 months later showing an exacerbated bend of the spinal cord at the T5/6 level (A) with a marked lateral elongation to the left of the cord (B). The lateral part of the stretched cord showing herniation into the vertebral erosion at the lower T5 level (B***,*** arrow). Coronal (C) and axial (D) computed tomography scans showing that the erosion of the T5 segment (*arrow*) did not change during the period. No identifiable intervertebral disk herniation or vertebral bone spur around T5/6 was observed.

**Fig. 3:**
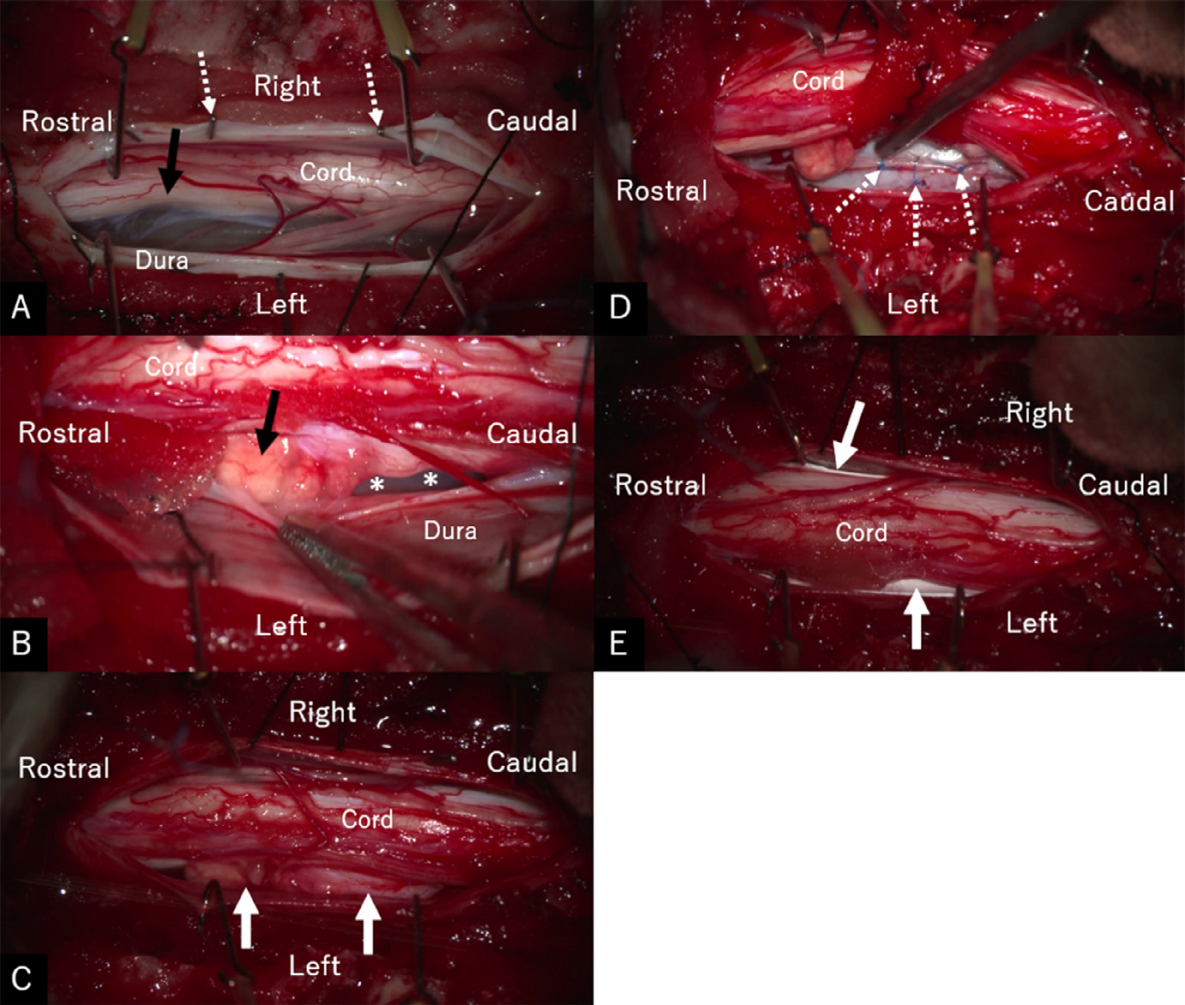
(A–E) Serial intraoperative images. (A) Vertical incisions in the dura mater and arachnoid membrane showing a bend in the thoracic cord at the T5/6 level (*arrow*). The dura mater and arachnoid membrane are reflected together using hemoclips (*dashed arrows*). (B) The spinal cord herniation is vertically long with a bulbous rostral part appearing like granulation tissues (*arrow*). (C) Following gentle, circumferential separation between the edges of the dura mater and herniated cord, the herniation entirely reduced (*arrows*). (D) The smooth-contoured dura defects sutured using 6-0 nylon threads (*dashed arrows*). (E) A piece of artificial dura mater (*arrows*) placed between the cord and sutured dura. *Asterisks* in (B): dura defect.

**Fig. 4:**
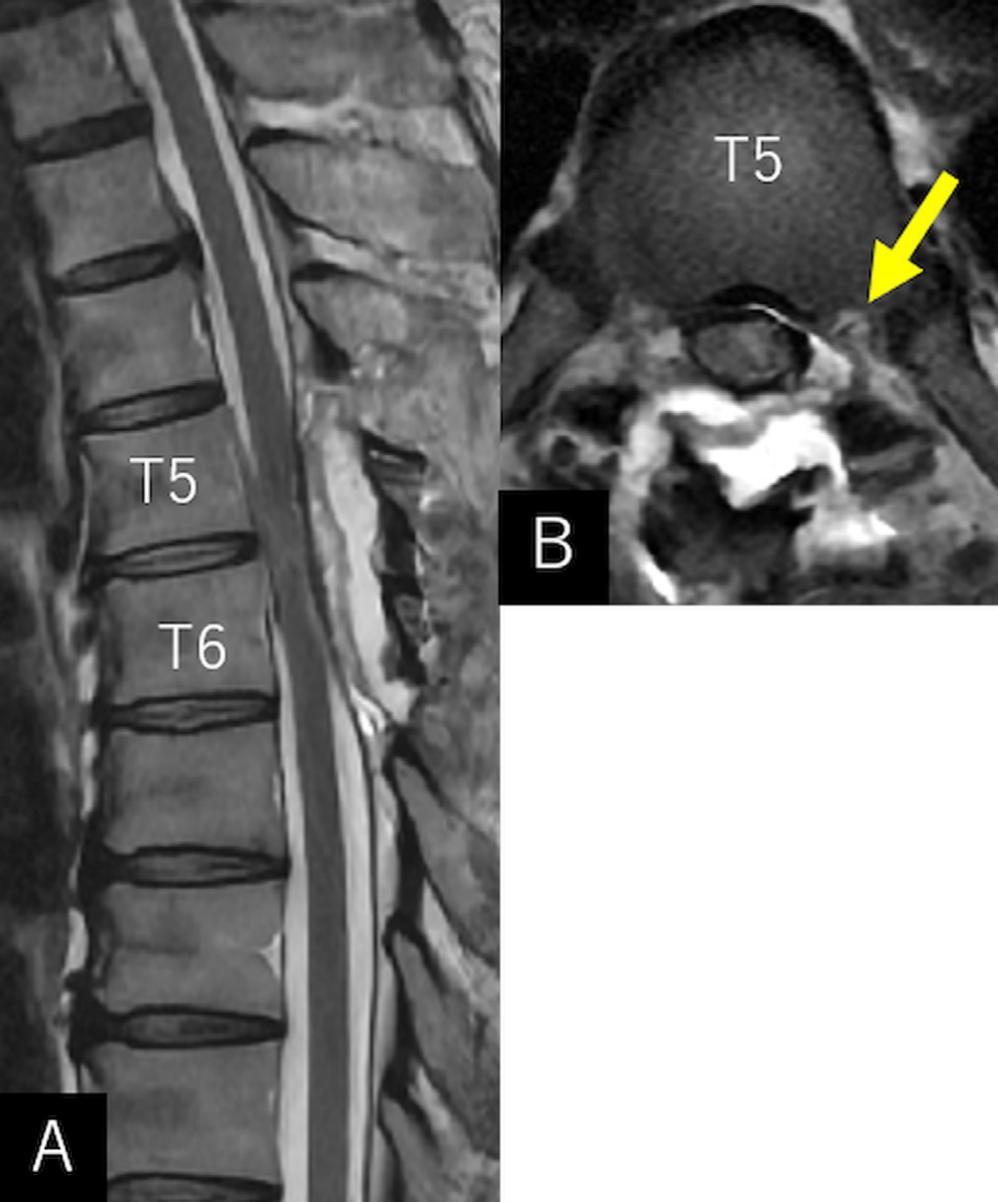
Sagittal (A) and axial (B) T2-weighted magnetic resonance imaging performed on the postoperative day 7 showing a restoration of herniated cord with complete separation between the dura sac and erosion in the lower T5 vertebra (*arrow*).

A 43-year-old man was referred to our hospital with a 6-month history of decreased sensation in the left lower extremity. His medical history was unremarkable, with no previous spinal trauma. At presentation, the patient exhibited slight motor weakness in the left quadriceps femoris, hamstring, and gluteal muscles and decreased pain sensation (5/10) below the left T7 dermatome. The knee jerk and Achilles tendon reflexes were promoted on the left side. Magnetic resonance imaging (MRI) of the spine revealed abnormal findings consistent with ITSCH at the T5/6 level, with localized ventral displacement of the cord (Figs. 1A and B). Computed tomography (CT) revealed a well-circumscribed, small vertebral erosion at the lower T5 just above the left facet joint (Figs. 1C and D). Despite a strong recommendation, the patient declined surgery. Instead, he requested that he be placed under observation with periodic MRI. However, the patient's symptoms gradually progressed over the next 3 years, and the motor weakness was 4/5 on the manual muscle test, with exacerbation of sensory loss. MRI performed at that time showed a marked lateral elongation of the cord at the T5/6 and apparent intravertebral cord herniation into the vertebral erosion. On CT, the erosion did not change during the 3 years. No identifiable intervertebral disk herniation or vertebral bone spur around T5/6 was noted (Fig. 2). Subsequently, the patient underwent microsurgical reduction of the ITSCH through T5-6 laminectomies. Intraoperatively, vertical incisions in the dura mater and arachnoid membrane revealed a bend of thoracic cord at the T5/6 (Fig. 3A). The ITSCH was vertically long and had a bulbous rostral part with the appearance of granulation tissue (Fig. 3B). Following gentle circumferential separation between the edges of the dura mater and herniated spinal cord, ITSCH reduction was achieved (Fig. 3C). The smooth-contoured margins of the dura defect were sutured using 6-0 nylon threads (Fig. 3D). In addition, a piece of artificial dura mater was placed between the cord and sutured dura for reinforcement (Fig. 3E). The patient's postoperative course was uneventful. The sensorimotor disturbances gradually improved. MRI performed on postoperative day 7 revealed restoration of the herniated cord with complete separation between the dura sac and erosion in the lower T5 vertebra (Fig. 4).

## Discussion

The etiology of ITSCH is not well understood and remains unclear. In the present case, ITSCH located at the T5/6 showed gradual symptomatic progression over 3 years. In addition, serial MRIs showed radiological progression of both spinal cord and intravertebral cord herniations. Therefore, we assumed that intravertebral spinal cord herniation was the main pathophysiology in the present case. Intravertebral spinal cord herniation is a rare ITSCH variant. To the best of our knowledge, only 1 case has been documented to date [8].

In our case, the herniated cord had an anomalous, bulbous portion, in addition to vertebral erosion where the lateral portion of the ITSCH was lodged. The erosion showed a stationary appearance. In addition, the margins of the dura defect had smooth contours without degeneration or mechanical injury. Therefore, the present ITSCH may develop in association with congenital factors. The bulbous part of the cord showed similar appearance to a previous report documenting as “congenital transdural appendix of the spinal cord” [3]. Notably, more than two-thirds of the reported ITSCHs, as with our case, have occurred at the intervertebral disc level, suggesting that a dynamic factor may contribute to the etiology [4]. Given that the affected cord showed a marked lateral elongation for 3 years, certain acquired factors also could have contributed to the progression of the present ITSCH. However, the correlations among cord elongation, vertebral erosion, and dynamic factors acting between them are elusive. The etiology of ITSCH may be multifactorial [7]. Commonly, ITSCHs are a progressive pathology. Physicians and radiologists must be aware that an ITSCH requires prompt diagnosis and surgical reduction [1,[9], [10], [11]].

In conclusion, ITSCH is a progressive pathology, even if gradual, and requires prompt diagnosis and microsurgical reduction. Certain ITSCHs may be complicated by intravertebral cord herniation.

## Patient consent

The patient documented in the manuscript fully understood and agreed that the authors use the information materials of the patient in anonymized manner for possible publication in Radiology Case Reports.

## References

[1] Jesse CM, Gallus M, Beck J, Ulrich CT, Seidel K, Piechowiak E (2023). Idiopathic ventral spinal cord hernia: a single-center case series of 11 patients. Oper Neurosurg (Hagerstown).

[2] Ball BG, Luetmer PH, Giannini C, Morki B, Kumar N, Piepgras DG. (2012). Ventral “spinal epidural meningeal cysts” – not epidural and not cysts? Case series and review of the literature. Neurosurgery.

[3] Bartels RHMA, Brunner H, Hosman A, van Alfen N, Grotenhuis JA. (2017). The pathogenesis of ventral idiopathic herniation of the spinal cord: a hypothesis based on the review of the literature. Front Neurol.

[4] Brus-Ramer M, Dillon WP. (2012). Idiopathic thoracic spinal cord herniation: retrospective analysis supporting a mechanism of discogenic sural injury and subsequent tamponade. AJNR Am J Neuroradiol.

[5] Hunziker S, Örgel A, Tatagiba M, Adib SD. (2023). Case report: A vertebral bone spur as an etiology for spinal cord herniation: case presentation, surgical technique, and review of the literature. Front Surg.

[6] Shimizu S, Kobayashi Y, Oka H, Kumabe T. (2019). Idiopathic spinal cord herniation: consideration of its pathogenesis based on the histopathology of the dura mater. Eur Spine J.

[7] Tyagi G, ARP Bhat DI, Rao MB, Devi BI (2019). Duplication of ventral dura as a cause of ventral herniation of spinal cord- a report of two cases and review of the literature. World Neurosurg.

[8] Turel MK, Wewel JT, Kerolus MG, O'Toole JE (2017). Idiopathic thoracic transdural intravertebral spinal cord herniation. I Craniovertebr Junction Spine.

[9] Darbar A, Krishnamurthy S, Holsapple JW, Hodge CJ (2006). Ventral thoracic spinal cord herniation: frequently misdiagnosed entity. Spine (Philla Pa 1976).

[10] Groen RJ, Middel B, Meilof JF, de Vos-van de Biezenbos JB, Enting RH, Coppers MH (2009). Operative treatment of anterior thoracic spinal cord herniation: three new cases and an individual patent data meta-analysis of 126 case reports. Neurosurgery.

[11] Jiang Q, Gao G, Tao B, Gao H, Wang H, Wang P (2023). Thoracic anterior spinal cord herniation: treatment and prognosis outcome of seven patients. World Neurosurg.

[12] Antony J, Neriamparambil AJ, Ma N. (2020). Case report of an anterior thoracic myelomeningocele: a multidisciplinary approach to surgical management. World Neurosurg.

